# Supplementary material for: Consistency of Condom Use with Lubricants and Associated Factors Among Men Who Have Sex with Men in Ghana: Evidence from Integrated Bio-Behavioral Surveillance Survey
Source: Int J Environ Res Public Health. 2025 Apr 11;22(4):599. doi: 10.3390/ijerph22040599 (PMC12026881; doi:10.3390/ijerph22040599)
Supplement: Supplementary file 1 [file ijerph-22-00599-s001.zip › Supplementary Material S1.pdf]

Ghana AIDS Commission
4th Floor, Olympic House, Ridge-Accra.
Digital Address: GA-052-9270

P.O.Box CT 5169, Cantonments, Accra
Tel: +233-302 919259 / 0302 919260
info@ghanaids.gov.gh | www.ghanaids.gov.gh
: @ghanaids

In case of reply, the **Number** & **Date** of this letter should be quoted

Your ref:

Our ref: GAC/LB/188/297/01

21/10/2024

Mr. Ratif Abdulai
(Student Number: 223052905)
South African Medical Research Council
University of Johannesburg
Pan Africa Centre for Epidemics Research Extramural Unit

Permission to Utilize the Ghana Men's Study II Dataset

This letter confirms permission by the Ghana AIDS Commission (GAC) to use de-identified data from the Ghana Men's Study II for your doctoral research entitled **"Condom and Lubricant Distribution, Consistency of Correct Utilization, and Associated Factors Among Men who have sex with Men in Ghana – A Predictive Model for Addressing Covid-19 Impacts"** at the South African Medical Research Council/the University of Johannesburg Pan Africa Centre for Epidemics Research Extramural Unit under the supervision of Professor Refilwe Nancy Phaswana-Mafuya and co-supervised by Dr Edith Phalane.

This permission granted to you is in line with the Data Sharing and Data Processing Agreement signed between the GAC and the SAMRC/UJ PACER Extramural Unit. We look forward to your adherence to ethical standards and sharing your findings with GAC and stakeholders.

We also request that the GAC be duly acknowledged in all your study publications and other knowledge dissemination activities such as conference presentations.

I wish you all the best in your doctoral research.
Sincerely,

Dr. Kyeremeh Atuahene
Director-General

OFFICE OF THE PRESIDENT
REPUBLIC OF GHANA
